# Proteomic and In Silico Analyses Highlight Complement System’s Role in Bladder Cancer Immune Regulation

**DOI:** 10.3390/medicina61040735

**Published:** 2025-04-16

**Authors:** Tuğcan Korak, İbrahim Halil Baloğlu, Murat Kasap, Elif Damla Arisan, Gurler Akpinar, Serdar Arisan

**Affiliations:** 1 Department of Medical Biology, Faculty of Medicine, Kocaeli University, Kocaeli 41001, Türkiye; 2 Seyrantepe Etfal Health and Application Research Center, Department of Urology, Hamidiye Medical School, University of Health Sciences, Istanbul 34396, Türkiye; 3 Institute of Biotechnology, Gebze Technical University, Kocaeli 41400, Türkiye

**Keywords:** bladder cancer, complement system, prognosis, proteomics, tumor immune microenvironment

## Abstract

*Background and Objectives*: Bladder cancer (BLCA), intimately associated with the immune system, represents a substantial global health burden due to its high recurrence rates and limited therapeutic effectiveness. Although immunotherapy shows promise, challenges persist due to the lack of reliable therapeutic targets. This study aims to investigate potential immune-related biomarkers that could influence the tumor microenvironment in BLCA, using proteomic and in silico approaches. *Materials and Methods*: Tissue samples from BLCA patients (n = 27) and controls (n = 27) were collected from Şişli Hamidiye Etfal Training and Research Hospital. Proteomic analysis was performed by liquid chromatography/mass spectrometry (LC-MS)/MS to reveal the identities of differentially regulated proteins. Protein network analysis and hub protein detection were performed using Cytoscape (v.3.10.3), while functional annotation was carried out using EnrichR. The immunological analysis of hub proteins was performed in Sangerbox platform, and prognostic associations were evaluated through the Kaplan–Meier Plotter tool. *Results*: LC-MS/MS analysis identified 120 differentially regulated immune-related proteins. STRING analysis, using an immune response dataset (GO:0006955), highlighted the complement cascade as a significantly enriched pathway (*p* < 0.05). Proteins, namely C4A, CFB, C4B, C8B, CFH, CFI, C5, C4BPA, C3, and C2, that are known to play key roles in the complement system were identified. Immunological analysis with these proteins revealed the phenomena of immune infiltration and immune checkpoint gene associations (*p* < 0.05). Four hub genes—CFB, C4B, CFI, and C2—demonstrated a significant prognostic value for BLCA (*p* < 0.05). *Conclusions*: This study highlights the pivotal role of the complement system in the immune regulation of BLCA. CFI, C4A, and C4B emerged as potential target proteins for BLCA treatment, particularly in immunotherapy, for enhancing survival. Future research on these proteins and the complement system specifically focusing on BLCA may facilitate the development of targeted immunotherapies, ultimately improving treatment outcomes.

## 1. Introduction

Bladder cancer (BLCA), or urinary bladder cancer, ranks as the 10th most common cancer worldwide, with a steadily increasing incidence. Recent GLOBOCAN statistics revealed that BLCA constituted 3% of global cancer cases, with a notably higher prevalence in more developed countries [1]. Despite advancements in early diagnosis, robotic surgical techniques, chemotherapy and radiotherapy, BLCA remains a significant clinical challenge due to its high occurrence rate. Therefore, a deeper understanding of the molecular pathways involved in its development and metastasis is essential to develop more effective prevention strategies and address the persistent challenges in its management [1,2,3].

Ongoing research suggests that immunotherapy holds the most promise for developing the best-tailored treatments for BLCA [3,4]. Recognized as an immune-related tumor type, substantial progress in identifying immune biomarkers and correlating them with immune target proteins has been made with BLCA to enhance prognosis, diagnosis, and therapeutic strategies [5]. For instance, the commonly used BLCA therapy, Bacille Calmette–Guérin (BCG), exerts its effects by activating immune cells such as macrophages, T-cells, and neutrophils within the tumor immune microenvironment, promoting interleukin (IL) release to induce tumor cell death. However, BCG also leads to elevated PD-L1 expression in tumor cells, potentially contributing to resistance in some patients. In such cases, immune checkpoint inhibitors (ICIs) targeting the PD-L1/PD-1 axis provide an alternative treatment option, but their response rates remain limited to ~20% [6,7]. The lack of reliable predictive biomarkers hampers the effectiveness of ICIs, whether administered independently or alongside chemotherapeutic regimens [8]. Additionally, the cost, extended treatment duration, and inconsistent predictive value of PD-L1/PD-1 expression levels further complicate their clinical utility. These limitations underline the need for novel immune-related target proteins for BLCA treatment to address current clinical shortcomings and improve the efficacy of immunotherapeutic approaches [5,9].

Proteomic technologies, with liquid chromatography/mass spectrometry (LC-MS) recognized as the gold standard, provide comprehensive protein profiling and are powerful tools for discovering novel molecular targets [10,11]. Several studies in the literature have focused on biomarker discovery across various BLCA subtypes and different sample types using proteomic approaches [12,13,14,15]. However, to the best of our knowledge, there is a lack of proteomic studies specifically targeting immune-related biomarkers in BLCA. The present study aimed to identify immune-related biomarkers that may influence or be influenced by the tumor microenvironment (TME) in BLCA. An LC-MS/MS-based proteomic approach was used to compare protein samples prepared from tumor and control tissues. The identified proteins were analyzed in silico to explore their immune associations, and key hub genes were further evaluated for prognostic purposes. Insights into their roles in BLCA progression and their potential in determining clinical outcomes were scrutinized.

## 2. Materials and Methods

### 2.1. Study Design and Patient Population

Tissues were collected from patients undergoing a transurethral resection of a bladder tumor (TUR-BT) and a transurethral resection of the prostate (TUR-P) at the Urology Clinic of University of Health Sciences Şişli Hamidiye Etfal Training and Research Hospital. Tumor tissue samples were obtained from patients diagnosed with bladder transitional cell carcinoma, while non-tumorous bladder neck tissues were collected from patients without bladder tumors during TUR-P procedures. Tissue samples were snap-frozen in liquid nitrogen and stored at −80 °C until use.

Patients diagnosed with BLCA based on the clinical and pathological findings were included in the current study. Individuals with cystoscopic findings suggestive of atypical bladder tumors, a history of recurrent bladder tumors, or concomitant malignancies were excluded from the study. Statistical analyses were conducted to confirm that the control and patient groups were normalized in terms of demographic and clinical variables, ensuring that observed differences in proteomic profiles were not confounded by external factors. All statistical analyses were performed using IBM SPSS Statistics for Windows, version 29.0 (IBMflrö Corp., Armonk, NY, USA). The Shapiro–Wilk test was used to assess normality assumption. Inter-group comparisons were made using the independent samples *t*-test, while associations between categorical variables were analyzed with the Chi-square test. A *p*-value of <0.05 was considered statistically significant. Sample size determination was performed using the G-Power 3.1 software, based on an effect size of 0.80, α error of 5%, and a statistical power of 80%, indicating the need for at least 26 patients in each group (tumor and non-tumor). The final analysis included 27 tumor tissue samples and 27 non-tumorous tissue samples. Among the 27 BLCA patients included in the study, CIS was present in 22.2% of cases. Tumor sizes ranged from 0.5 cm to 6 cm, and the cohort included a mix of TaLG, T1HG, T2HG, and TAHG tumors, covering both low- and high-grade disease. Ta, T1, and T2 represent tumor stages, while low-grade (LG) and high-grade (HG) classifications indicate tumor differentiation levels. According to postoperative tumor pathology results, 6 patients had muscle-invasive bladder cancer, while 21 had non-muscle-invasive bladder cancer. The micropapillary variant was present in 1 patient, and the pathology of 1 patient was reported as choroid carcinoma. CIS positivity was observed in 6 patients, whereas 21 were CIS-negative. Among the 27 patients, 11 had TaHG, 2 had T1HG, and 8 had T2HG tumors. The demographic and clinical characteristics of the BLCA patients are shown in Table 1.

The average follow-up duration of the 27 patients in the tumor group was calculated as 13.33 ± 5.84 months. During the follow-up period, 4 patients passed away. Recurrences were observed in 10 patients. Among the patients with a recurrence, only one showed progression to a higher grade. As the patients were under close follow-up, curative treatment effects were not assessed.

Informed consent was obtained from all participants, and the study was carried out in compliance with the Helsinki Declaration. Ethical approval was granted by the Clinical Research Ethical Board of University of Health Sciences Şişli Hamidiye Etfal Training and Research Hospital (approval number: 2024/2697).

### 2.2. Protein Extraction and Pool Formation

Tissue samples were washed with PBS to remove residual blood and then dissected into small pieces using a scalpel. RIPA buffer (Thermo Fisher Scientific, Waltham, MA, USA, 89900) and a protease inhibitor cocktail (Merck, Darmstadt, Germany, 11697498001) were added to the tissue samples. The tissues were incubated on ice for 30 min, and 0.2 and 0.5 mm stainless-steel beads (Next Advance, Troy, NY, USA) were added. The samples were then homogenized using a homogenizer (Next Advance Bullet Blender) at +4 °C. After homogenization, the samples were centrifuged at 15,000× *g* for 30 min, and the supernatant containing proteins was subjected to a Bradford assay (BioRad, Hercules, CA, USA) to measure protein concentrations. Protein pools were created from the tumor and control samples by combining equal amounts of protein from each sample, encompassing all BLCA tumor subtypes to provide a comprehensive analysis. The protein concentrations of each pool were measured and then verified by SDS-PAGE electrophoresis.

### 2.3. Enzymatic Digestion and LC-MS/MS Analysis

The digestion of pooled protein samples was performed using the filter-aided sample preparation kit protocol (Expedeon, Cambridge, UK, 44250). After digestion, peptides were eluted, concentrated using a SpeedVac (Eppendorf, Leipzig, Germany, 22331), and resuspended in 20 µL 0.1% formic acid (FA) for LC-MS/MS analysis. Peptide concentrations were measured using a Qubit 4 Fluorometer (Invitrogen, Carlsbad, CA, USA, Q33226). For LC-MS analysis, they were equalized between the tumor and control pools.

The digested peptides were analyzed using LC-MS/MS on an Ultimate 3000 RSLC nanosystem (Dionex, Thermo Fisher Scientific) coupled with a Q Exactive mass spectrometer (Thermo Fisher Scientific). Peptides were separated on a C18 reversed-phase column (Thermo Fisher Scientific, Acclaim PepMap RSLC, 75 μm × 15 cm, Waltham, MA, USA) after preconcentration and desalting. A linear gradient from 6% to 90% solvent B was used, with varying percentages over time at a flow rate of 300 nL/min. Data-dependent acquisition was performed on the top 10 precursor ions within a mass range of 400–2000 *m*/*z*, with a resolution of 70,000. The data were analyzed using SEQUEST in Proteome Discoverer, with a peptide mass tolerance of 10 ppm and an MS/MS mass tolerance of 0.2 Da. Cysteine carbamidomethylation was set as a fixed modification, while methionine oxidation and asparagine deamination were set as variable modifications [16]. Peptide identifications with >95% probability and protein identifications with >99% probability (at least two peptides) were accepted. A false discovery rate (FDR) threshold of 0.01 for strict and 0.05 for relaxed criteria was applied. Proteins showing a two-fold significant expression difference and identified with at least two unique peptides between tumor and control groups were defined as differentially regulated proteins (DRPs).

### 2.4. Functional Annotation and Pathway-Based Analysis of Immune-Associated Proteins

Following the identification of DRPs through LC-MS/MS analysis, the homo sapiens immune response pathway (GO:0006955) was created using the search tool for the Retrieval of Interacting Genes/Proteins (STRING) database (confidence level: 0.400). The DRPs were then intersected with immune response-related proteins in Cytoscape (v. 3.10.3). The common proteins between both groups were functionally annotated by performing reactome pathway analysis via the Enrichr platform. Statistical significance was set to *p* < 0.05, and the pathways were ranked based on their *p*-values [17]. The top 10 key proteins within the intersected network were selected using CytoHubba’s maximal clique centrality (MCC) algorithm, which is recognized for its high accuracy in identifying essential proteins within protein–protein interaction (PPI) networks by detecting high-centrality nodes and strong functional interactions [18,19].

### 2.5. Immunological Analysis

To evaluate the relationship between hub proteins and overall immune infiltration in the BLCA TME, ImmuneScore association analysis was performed. Expression data for each key protein specific to BLCA were retrieved from the TCGA TARGET GTEx dataset and used for the calculation of ImmuneScores, quantifying overall immune infiltration. Subsequent TIMER analysis was conducted using the same dataset to explore the relationship between the expression of hub proteins and the infiltration of six main immune cell types, B cells, CD4+ T-cells, CD8+ T-cells, neutrophils, macrophages, and dendritic cells, as defined in previous studies [20,21]. This analysis specifically assessed the associations between these immune cell populations and hub protein expressions within the BLCA TME. To further deepen immunological associations, the relationship between hub gene expressions and immune checkpoint proteins (ICPs) was evaluated. Pearson correlation analysis was performed between the expression levels of each hub gene and 60 ICP marker genes (24 inhibitory and 36 stimulatory), available in the Sangerbox (v.3.0), using the BLCA TCGA dataset. All immunological analyses were conducted using the Sangerbox platform (v.3.0) [22], and correlations with *p*-values < 0.05 were considered statistically significant.

### 2.6. Prognostic Analysis of Hub Genes

The relationship between hub gene expression levels (categorized as high and low) and prognostic outcomes was analyzed using the overall survival (OS) module in the Kaplan–Meier (KM) Plotter tool (https://kmplot.com/analysis/, accessed on 9 April 2025). In the KM plots, the time variable represents the duration in months from the time of diagnosis to the occurrence of the event of interest [23]. A *p* value of <0.05 was considered the threshold for statistical significance.

### 2.7. Verification of LC-MS/MS Analysis via Western Blot

To verify the LC-MS/MS analysis results, Western blot analysis was performed following the protocol detailed in our previous study, with total protein normalization [24]. 14-3-3 beta/alpha was selected for validation due to the widespread downregulation of 14-3-3 protein family members observed in BLCA, making it a representative candidate for confirming the reliability of our proteomic findings. For the validation of 14-3-3 beta/alpha, a significantly regulated protein between the BLCA and control groups, Western blot analysis was conducted using an 14-3-3 beta/alpha primary antibody (Cell Signaling, Trask Lane Danvers, MA, USA, #9636) and a goat anti-rabbit secondary antibody (Bio-Rad, Hercules, CA, USA, #170-5046).

## 3. Results

### 3.1. Study Cohort and Immune Correlated LC-MS/MS Data Analysis

**Figure 1:**
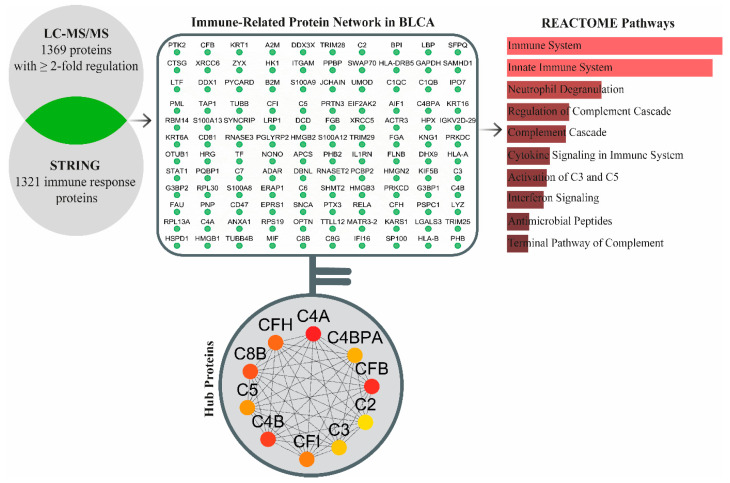
The immune-related hub proteins determined via combinational analysis of LC-MS/MS data and data retrieved from STRING. Statistically significant reactome annotations (*p* < 0.05) are indicated in the bar graph. The top 10 central proteins within the network are represented by nodes ranging from red to yellow, with red indicating the highest centrality score and yellow indicating lower centrality.

Statistical analysis revealed no significant differences between BLCA and healthy participants regarding gender, age, smoking status, and concurrent malignancies (*p* > 0.05). The cohort provided a reliable basis for the comparison of BLCA patients and healthy controls. LC-MS/MS analysis identified a total of 2559 proteins, of which 1369 were differentially regulated between tumor and control tissues based on a two-fold regulation and the presence of at least two unique peptide criteria (Supplementary Table S1). Initial screening of the data using bioinformatic tools underlined the changes in immune response pathways. Therefore, immune response-related proteins were retrieved from the STRING database for comparison. Cross-checking of our data against dataset retrieved from STRING using Cytoscape revealed the presence of 120 common proteins. A functional annotation of the 120 proteins indicated that 10 pathways were significantly affected in tumor tissues: the immune system (*p* = 1.200 × 10^−42^), innate immune system (*p* = 4.878 × 10^−41^), neutrophil degranulation (*p* = 2.129 × 10^−22^), regulation of complement cascade (*p* = 5.361 × 10^−17^), complement cascade (*p* = 2.059 × 10^−16^), cytokine signaling in immune system (*p* = 8.811 × 10^−14^), activation of C3 and C5 (*p* = 2.863 × 10^−13^), interferon signaling (*p* = 9.786 × 10^−13^), antimicrobial peptides (*p* = 2.719 × 10^−10^), and terminal pathway of complement (*p* = 3.946 × 10^−10^). Following identification of 120 immune-related DRPs in BLCA, the central proteins within this PPI network constructed from these DRPs were determined using the maximal clique centrality (MCC) algorithm. The top 10 key proteins identified were Complement 4A (C4A), Complement Factor B (CFB), Complement 4B (C4B), Complement C8 Beta Chain (C8B), Complement Factor H (CFH), Complement Factor I (CFI), Complement C5 (C5), Complement 4 Binding Protein Alpha (C4BPA), Complement C3 (C3), and Complement C2 (C2) (Figure 1). The hub proteins determined in this study were upregulated in tumor tissues in comparison to healthy control tissues (Table 2).

### 3.2. Association of Hub Proteins with Tumor Immune Microenvironment

**Figure 2:**
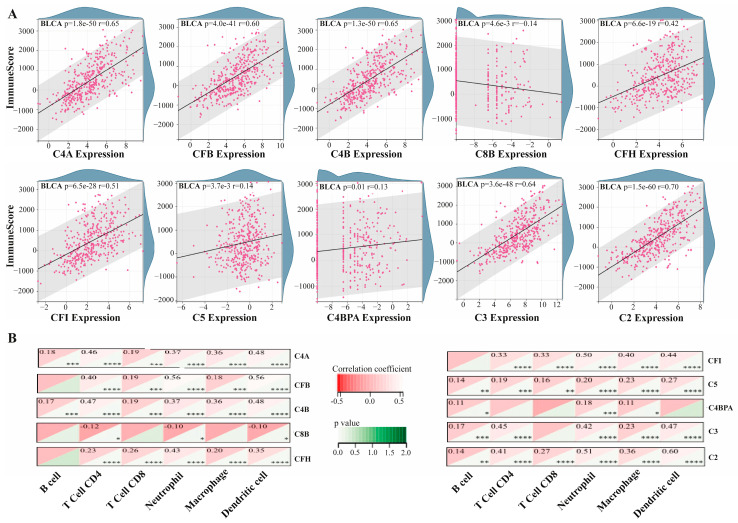
Correlation between hub protein expression and immune infiltration in BLCA. (**A**) Significant associations between the expression of each hub protein and ImmuneScore in a BLCA tumor microenvironment (*p* < 0.05). Data points are represented by pink dots on the scatter plot, while the surrounding density plot illustrates data distribution. (**B**) Association of hub protein expression with the infiltration of six major immune cells (B-cells, CD4+ T-cells, CD8+ T-cells, neutrophils, macrophages, and dendritic cells) in BLCA. In the heatmap, red areas represent the correlation coefficient, while green areas indicate *p*-values (* *p* < 0.05, ** *p* < 0.01, *** *p* < 0.005, **** *p* < 0.001).

ImmuneScore association analysis was performed to evaluate the link between hub proteins and overall immune infiltration in the BLCA tumor microenvironment. Assessment of ImmuneScore correlations for each hub protein revealed the presence of a strong correlation between immune infiltration and the BLCA tumor microenvironment. The correlation values for each protein was determined to be *p* = 1.8 × 10^−50^, r = 0.65 for C4A; *p* = 4.0 × 10^−41^, r = 0.60 for CFB; *p* = 1.3 × 10^−50^, r = 0.65 for C4B; *p* = 4.6 × 10^−3^, r = −0.14 for C8B; *p* = 6.6 × 10^−19^, r = 0.42 for CFH; *p* = 6.5 × 10^−28^, r = 0.51 for CFI; *p* = 3.7 × 10^−3^, r = 0.14 for C5; *p* = 0.01, r = 0.13 for C4BPA; *p* = 3.6 × 10^−48^, r = 0.64 for C3; and *p* = 1.5 × 10^−60^, r = 0.70 for C2. Except C8B, all of the hub proteins displayed positive correlations with immune infiltration (Figure 2A). Beyond the relationship between hub genes and immune infiltration, TIMER analysis revealed the associations between each hub protein and the infiltration of six immune cell types. Notably, each hub protein showed a statistically significant correlation with three or more of these infiltrations. Interestingly, C4A, C4B, C5, and C2 showed significant correlations with all immune cell infiltration subtypes, while CFB, CFH, CFI, and C3 were significantly associated with five immune cell infiltrations. The least significant associations were observed for C8B with T-cell CD4, neutrophil, and dendritic cell (DC) infiltrations, and for C4BPA with B-cell, neutrophil, and macrophage infiltrations. A negative correlation was found only for C8B (r < 0) (Figure 2B).

### 3.3. Association Between Hub Gene Expression and ICP Markers

**Figure 3:**
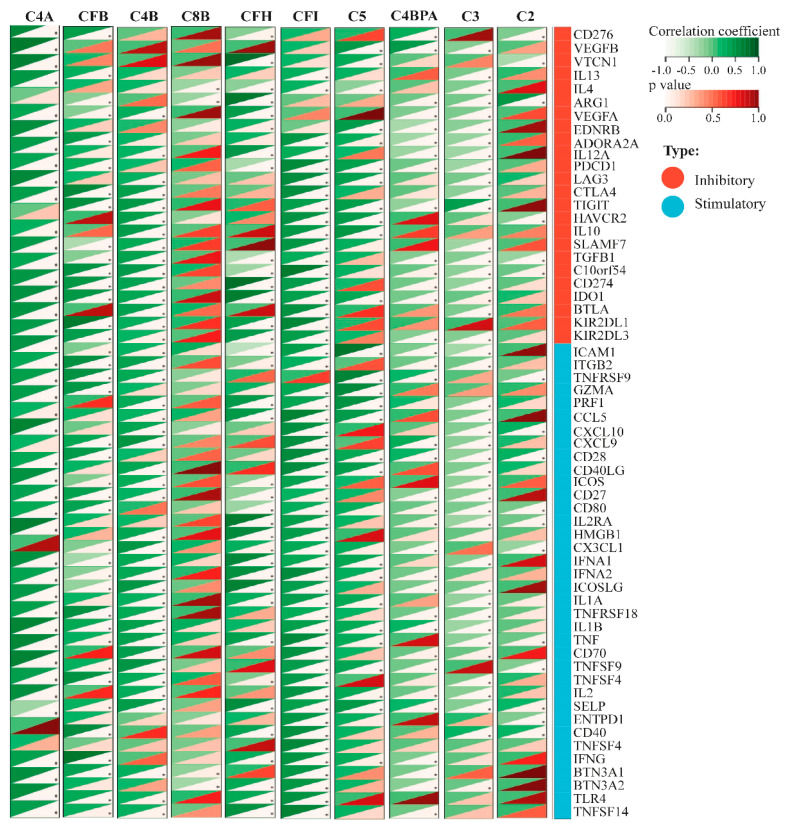
Correlation of hub genes with immune checkpoint (ICP) markers. The heatmap illustrates the significant associations (* *p* < 0.05) between hub gene expressions (C4A, CFB, C4B, C8B, CFH, CFI, C5, C4BPA, C3, and C2) and 60 ICP marker genes. Green represents the correlation coefficients, while red denotes the corresponding *p* values. The red bar within the heatmap signifies the 24 inhibitory ICP markers, whereas the blue bar represents the 36 stimulatory ICP markers.

Immune checkpoint protein (ICPs) genes play crucial roles in regulating immune cell infiltration and shaping the effectiveness of cancer therapies [20]. To explore the relationship between hub gene expression and immune infiltration, we examined their associations with a panel of 60 ICP marker genes. Several hub genes displayed statistically significant stimulatory and inhibitory associations with ICP markers (*p* < 0.05). For instance, C4A, C3, C4B, CFB, CFH, C4BPA, C5, C2, and C8B displayed 54, 53, 43, 42, 40, 37, 35, 27, 17, and 54 associations with ICP markers, respectively. Notably, C4A and CFI exhibited the highest number of significant associations, while C8B demonstrated the fewest. All significant correlations were positive (r > 0) (Figure 3).

### 3.4. Prognostic Value of Hub Genes

**Figure 4:**
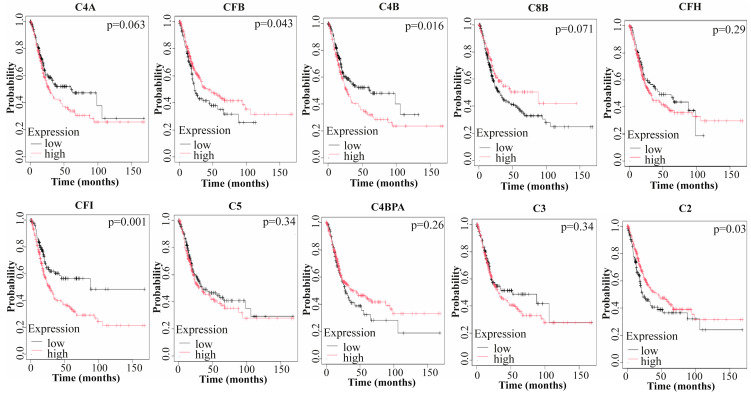
Prognostic significance of hub genes. Kaplan–Meier plots illustrate that four hub genes—CFB, C4B, CFI, and C2—exhibit statistically significant associations with overall survival (OS) (*p* < 0.05). Red lines indicate high gene expression, while black lines represent low gene expression.

In BLCA, Kaplan–Meier plots demonstrating the prognostic significance of hub genes revealed that four hub genes—CFB, C4B, CFI, and C2—had statistically significant prognostic outcomes (*p* < 0.05). Among these, high expression levels of CFB and C2 were associated with favorable overall survival (OS), whereas the increased expression of C4B and CFI was linked to a shorter lifespan (Figure 4).

### 3.5. Validation of Proteomic Analysis

The results of Western blot demonstrated that 14-3-3 beta/alpha protein expression was significantly reduced by nearly 27.5-fold in BLCA samples compared to the control group (*p* < 0.05) (Supplementary Figure S1). This result is consistent with the proteomic analysis, further confirming the observed downregulation trend of 14-3-3 proteins in BLCA. These findings reinforce the robustness of our proteomic data.

## 4. Discussion

Bladder cancer (BLCA) continues to represent a significant global health challenge due to its aggressive nature, high recurrence rates, and limited treatment options. The development of reliable biomarkers is crucial for improving patient outcomes, and immune-related markers, in particular, hold great promise for advancing prognostic and therapeutic strategies in BLCA [2,3,25]. Taking these insights into account, this study aimed to identify key immune-related markers associated with BLCA by integrating proteomic and in silico approaches, correlating these markers to TME and ICPs, and assessing their prognostic significance to lay the groundwork for advancing immunotherapeutic approaches.

Through proteomic analysis, significantly regulated proteins in BLCA were identified and linked to the immune response pathways. Functional annotation of the resulting protein interaction network highlighted the complement cascade as a central pathway. The complement system, a key component of immune defense, plays a complex role in cancer, influencing immune cells, cancer cells, and angiogenesis. While it participates in immune surveillance and provides anti-tumor defense through mechanisms like complement-dependent cytotoxicity, major findings indicate that complement system activation predominantly contributes to pro-tumor effects in certain cancers. Although the dual role of the complement system may vary across different cancer types, its exact function has yet to be fully clarified [26,27]. In certain carcinomas, such as prostate, thyroid, and adrenocortical cancers, the complement system has been found to exert protective effects. However, in others, including uterine, stomach, and colon carcinomas, it has been shown to promote tumor aggressiveness. Its role in BLCA progression remains unclear [27]. For instance, one study reported that increased expression of the complement system-related protein CD46 was linked to enhanced invasive characteristics of BLCA tumor cells [28], while another study pointed to the protective effects of this system in BLCA [29]. Our findings suggested that the aberrant regulation of complement-related proteins might influence tumor progression in BLCA, further supporting the idea that this system could play a significant role in modulating tumor progression in BLCA. Therefore, the complement system represents a potential therapeutic target for enhancing cancer immunotherapy outcomes in BLCA.

The complement system, an important part of the innate immune system, consists of over 50 soluble and membrane-bound proteins (including C1-C9, C4BPA, CFI, and CFH). It plays a critical role in immune defense, inflammation, and tissue homeostasis. The system is activated through three main pathways, namely classical, lectin, and alternative, ultimately leading to the formation of the membrane attack complex (MAC), which induces cell lysis. Beyond its role in pathogen elimination, complement activation in cancer can be hijacked to support tumor progression, influencing immune responses and fostering inflammatory environments that promote tumor growth and metastasis. Despite its well-established role in immune responses, the precise involvement of the complement system in BLCA remains incompletely understood and demands further investigation [30,31,32].

Within the immune-related protein network in BLCA, which is heavily annotated with the complement system, we identified hub proteins that may have biomarker potential, aiming to detect those that could play crucial roles in immune response regulation in BLCA. Each identified hub protein—C4A, CFB, C4B, C8B, CFH, CFI, C5, C4BPA, C3, and C2—was associated with the complement system in accordance with the functional annotation results, and all were found to be upregulated in BLCA. Such upregulation might reflect an imbalance in the immune system, where the complement system, instead of offering protective anti-tumor effects, may inadvertently support tumor growth through processes like proliferation, migration, and epithelial–mesenchymal transition (EMT) [27]. In line with our findings, studies have shown that C3-associated complement system activation triggers processes such as tumorigenesis, EMT, and angiogenesis in cervical and ovarian cancers [33,34], while tumor tissue deposition has been observed in glioblastoma and lung cancer [35,36]. C5-correlated components have been implicated in enhancing tumor progression and invasiveness in cervical, ovarian, and colon cancers [34,37,38]. Furthermore, studies associating C1, C4, and C5 with cancer have demonstrated tumor tissue deposition effects in various cancer types, including breast, thyroid, and ovarian cancers. In cutaneous squamous cell carcinoma (sCC), increased tumor tissue expression of CFH has been observed and suggested as a potential biomarker. Consistent with our study, analysis of BLCA patient samples revealed elevated CFH concentrations in the urine of BLCA patients [26,39]. As evidenced, research on BLCA and its association with the complement system remains insufficient. However, our data align with findings from other malignancies, where similar complement system components, including our identified hub proteins, contribute to tumor progression.

In certain cancers, complement components contribute to tumor progression by facilitating the recruitment of myeloid-derived suppressor cells (MDSCs) and promoting immunosuppressive signaling, as observed in colorectal and breast cancer. However, in other contexts, such as in cSCC and melanoma, they may support anti-tumor responses by promoting immune cell infiltration, including the increased presence of effector T-cells and dendritic cells, which contribute to a more robust anti-tumor immune response [27,40]. Many studies in this regard have demonstrated that complement pathways influence tumor fate by modulating the TME [41]. Building upon this evidence, we performed an immune infiltration analysis in BLCA, correlating the complement hub proteins with immune infiltration profiles. The results showed that all hub proteins, except C8B, were positively associated with overall immune infiltration. Further analysis revealed that eight hub proteins exhibited positive correlations with a majority of six main immune cell types, while C8B showed a negative correlation, and C4BPA had the weakest association. The negative correlation between C8B and tumor immune infiltration in BLCA suggests a role in suppressing immune responses within the TME. C8B is a component of the membrane attack complex (MAC), which plays complex roles in cancer. Beyond its classical cytolytic function, MAC can activate various signaling pathways in tumor cells that promote survival and proliferation. These processes may also involve the suppression of immune cell infiltration, contributing to immune evasion and the creation of an immunosuppressive TME. On the other hand, the upregulation of the other nine complement hub proteins in BLCA tumors likely leads to immune cell recruitment, yet this may not result in effective anti-tumor responses. Instead, the activated complement system may create an immunosuppressive TME, where infiltrating immune cells are functionally inhibited [42]. Supporting this notion, several studies have shown that complement components such as C3a and C5a mediate tumor-promoting responses by modulating immune cell functions, recruiting MDSCs, and shifting immune responses toward immunosuppressive T-helper-2 (TH2) cells and regulatory T-cells (Tregs). Moreover, complement activation supports cancer cell survival by maintaining stemness and promoting proliferation through C3a–C3aR signaling. These processes contribute to a TME that favors malignancy progression. Additionally, C5a regulates the self-renewal of tissue-resident pulmonary alveolar macrophages, which suppress anti-tumor immunity [42,43]. It has also been suggested that C4A promotes tumor growth through its interaction with tumor-associated macrophages [44]. Thus, the upregulated, complement system-associated hub proteins in BLCA may paradoxically support tumor progression by modulating immune responses in the TME.

ICP genes are critical regulators of immune cell infiltration and have become key targets in cancer immunotherapy, particularly in BLCA, where their modulation holds significant therapeutic potential. While the enhanced efficacy and tolerance of immune checkpoint inhibitors (ICIs) have been established in BLCA patients, further research is still needed to optimize treatment strategies for this patient population [20,45]. In the current study, the hub genes—particularly C4A and CFI—showed significant positive correlations with the expression of most. C4A may promote tumor growth by interacting with tumor-associated macrophages, potentially enhancing immune evasion and inflammation [44,46]. CFI could contribute to tumor progression by inhibiting complement activation, possibly creating a protective microenvironment that supports tumor survival and invasiveness [46,47]. Beyond their known functions, our study suggests that in BLCA, C4A, and CFI may also drive cancer progression by modulating immune-related checkpoints, offering additional insight into their role in tumor immunology.

In OS analyses of immune-related hub genes in BLCA, it was observed that high CFB and C2 expression were correlated with longer survival, while C4B and CFI were linked to shorter survival. Although there are no experimental prognostic studies on these hubs in BLCA, a study on renal cell carcinoma (RCC) found that C4B suppression was associated with increased survival in patients receiving cytokine therapy, and a deficiency of C4B in stage IV RCC patients was linked to better survival [48]. On the other hand, elevated expression levels of CFI have been reported to correlate with tumor invasiveness and poor prognosis in cSCC, breast cancer, and glioma [47]. Additionally, protective effects of C2 have been demonstrated in melanoma [49]. While our findings align with these findings, the role of CFB remains controversial, with studies reporting anti-tumor effects in lung adenocarcinoma [50], whereas its expression is associated with poor prognosis in prostate cancer [51]. In another comprehensive study, the complement system was found to have protective roles in certain cancers, such as mesothelioma, sarcoma, and hepatocellular carcinoma, while high complement activation was associated with poor prognosis in melanoma, glioma, and lung cancer. Similarly to our findings, this large-scale database-driven study did not identify a clear prognostic trend between complement expression and OS in BLCA [52]. The discrepancies could be attributed to differences in cancer types and the co-expression patterns of complement system components, which could result in varying effects on tumor progression [53].

The overexpression of membrane-bound complement regulatory proteins and soluble inhibitors enables tumor immune evasion, contributing to therapy resistance and poor prognosis. Elevated CD55 and CD59 levels are linked to reduced survival in multiple cancers, including BLCA, aligning with our findings that key complement-related proteins drive BLCA progression through their interaction with the TME [30]. A meta-analysis of 50 complement-related genes across 30 cancers reported high expression of classical pathway components, such as C4A and C2, including in BLCA, consistent with our results. However, while our study found C8B upregulation in BLCA, the meta-analysis reported downregulation of C8B and other complement regulators in BLCA, possibly due to sample differences, tumor heterogeneity, or variations between transcriptomic and proteomic analyses [52]. Additionally, C3 was consistently overexpressed across cancers, supporting its tumor-promoting role, as evidenced by reduced tumor growth in C3-deficient mice in lung, breast, and cutaneous squamous cell carcinoma models [30,54,55]. Consistent with findings in various cancers, our results reinforce the intricate relationship between the complement system and tumor progression in BLCA, highlighting the necessity of deciphering its complex regulatory network to advance our understanding of cancer biology and optimize therapeutic strategies.

Modulation of the complement system in BLCA influences tumor progression and immunotherapy efficacy by regulating the TME. Targeting complement activation may reduce immunosuppressive cell recruitment, enhancing anti-tumor responses. Clinically, C3 and C5 inhibitors have been explored for their potential to improve immune checkpoint blockade efficacy and limit tumor-associated inflammation. Combining complement-targeting therapies with BCG vaccination and immune checkpoint inhibitors could yield synergistic effects, improving patient outcomes. Beyond therapeutic applications, complement-related proteins could serve as valuable biomarkers for risk stratification, early detection, and treatment response monitoring. The inclusion of complement hub proteins (e.g., CFB, C4B, CFI, and C2) in biomarker panels could provide clinically relevant prognostic insights and support personalized treatment strategies. Additionally, droplet digital PCR (ddPCR) enables targeted pathogen detection, including *P. somerae*, linked to BLCA. Integrating PCR-based diagnostics with complement-targeting strategies may enhance BLCA screening, patient stratification, and treatment optimization, leading to improved clinical outcomes [30,56,57].

Beyond the complement system, various immune-related mechanisms have been implicated in BLCA progression and immune regulation, aligning with our findings. Previous studies have demonstrated that neutrophil degranulation facilitates BLCA progression by promoting immune evasion, tumor growth, and angiogenesis through neutrophil extracellular trap (NET) formation. Increased NET accumulation and impaired DNase I-mediated degradation have been shown to create a tumor-promoting microenvironment, enhancing tumor adhesion, migration, and immune resistance, which supports our observations and further underscores the role of innate immune mechanisms in BLCA [58]. Similarly, cytokine signaling, particularly through IL-6, IL-8, and tumor necrosis factor-α, has been identified as a key contributor to BLCA pathogenesis by fostering chronic inflammation and immune cell recruitment, findings that are in concordance with our study [59]. Interferon signaling defects have also been reported to play a crucial role in BLCA immune escape by impairing antigen presentation and diminishing immunotherapy efficacy, further highlighting the complex interplay between immune pathways in BLCA regulation [60]. Additionally, antimicrobial peptides such as defensins have been shown to exhibit dual functions in BLCA, acting as tumor suppressors by modulating immune responses or as tumor promoters by fostering a pro-inflammatory and immunosuppressive environment, consistent with our findings [61]. These studies collectively reinforce the notion that a broader exploration of innate immune pathways in BLCA may provide valuable insights into immunotherapy resistance and potential therapeutic targets.

Smoking is a well-established risk factor for BLCA that has been shown to influence the TME and immune-related protein expression. Chronic inflammation and oxidative stress induced by smoking can alter cytokine release, immune cell infiltration, and complement activation, potentially impacting the expression of complement-associated proteins identified in our study. Additionally, smoking-related epigenetic modifications and immune suppression may contribute to variability in immune responses among BLCA patients [62,63]. Investigating the relationship between smoking and immune-related protein expression could offer valuable insights into its role in BLCA pathophysiology and immune regulation.

While this study provides valuable insights into the role of complement-associated proteins in BLCA, certain limitations should be acknowledged. The sample size remains a constraint, and future studies with larger cohorts are needed to enhance statistical power, generalizability, and robustness of the findings, as well as to enable subtype-specific proteomic analyses for a deeper understanding of molecular differences. Another consideration is the impact of smoking status and other clinical factors on immune-related protein expression, which should be explored in future studies to determine their potential role in complement regulation within the BLCA microenvironment. Knockdown or overexpression studies using siRNA, shRNA, or CRISPR/Cas9 could clarify the role of hub proteins in tumor growth, immune evasion, and complement activation. Additionally, ELISA validation of hub proteins in an independent patient cohort would enhance result robustness. Functional assays in BLCA cell lines or organoid models, including C3 or C5 inhibition, could further reveal how complement activation impacts tumor progression, immune infiltration, and immunotherapy response. Additionally, future studies should explore the applicability of proteomic assays in urine samples as a non-invasive approach for early detection and BLCA monitoring.

One important limitation of this study is the absence of validation in an independent patient cohort. While the discovery-phase findings are promising, independent validation is essential to confirm their reproducibility, reliability, and clinical relevance. Such a validation cohort would enable assessment of the identified biomarkers across a broader, more diverse population, help to eliminate potential sample-specific biases, and strengthen the generalizability of the findings. Future studies incorporating larger, independent cohorts are necessary to verify the diagnostic and prognostic value of the identified complement-related proteins and to evaluate their applicability in clinical decision-making and therapeutic stratification in BLCA. Addressing these aspects in future research will refine our understanding of the complement system’s role in BLCA and its potential as a therapeutic target.

## 5. Conclusions

In conclusion, our study highlighted the pivotal role of the complement system in BLCA, emphasizing its dual involvement in tumor progression and immune modulation within the TME. Among the identified immune-related hub proteins—CFI, C4A, and C4B—these key players stand out for their significant implications in BLCA biology. CFI, with its strong relationships with immune infiltration, immune checkpoints, and prognosis, emerges as a promising biomarker for immunotherapy and patient survival. C4A, as a central complement regulator, plays a crucial role in immune modulation, while C4B’s robust relationship with immune regulation further underlines its prognostic value. These complement-related proteins offer new insights into the immune landscape of BLCA, making them valuable candidates for future therapeutic strategies. Future research should focus on validating these biomarkers in clinical cohorts and exploring their full potential in personalized treatment strategies for BLCA, enhancing both therapeutic outcomes and prognostic accuracy.

## Figures and Tables

**Table 1:** Demographic and clinical properties of BLCA patients and healthy controls.

BLCA Patients	
Gender, n (%)	
Male	15 (55.6)
Female	12 (44.4)
**Age (mean ± SD), years**	68 ± 10.5
**Tumor diameter (mean ± SD), cm**	3.07 ± 1.8
**Recurrence, n (%)**	
Observed	9 (33.3)
Not observed	18 (66.7)
**Smoking, n(%)**	
Present	25 (92.6)
Absent	2 (7.4)
**Concurrent malignancy, n (%)**	
Present	0 (0)
Absent	27 (100)
**Healthy Controls**	
**Gender, n (%)**	
Male	16 (59.3)
Female	11 (40.7)
**Age (mean ± SD), years**	63.4 ± 8.5
**Smoking, n (%)**	
Present	19 (70.4)
Absent	8 (29.6)
**Concurrent malignancy, n (%)**	
Present	0 (0)
Absent	27 (100)

**Table 2:** Expression levels of the hub proteins in BLCA tumors compared to healthy control tissues.

Gene Name	Uniprot ID	Expression Fold Change
C4A	P0C0L4	4.9
CFB	P00751	3.7
C4B	P0C0L5	6.2
C8B	P07358	3.4
CFH	P08603	10.1
CFI	P05156	3.1
C5	P01031	2.8
C4BPA	P04003	5.2
C3	P01024	5.5
C2	P06681	2.1

## Data Availability

The data generated in this study are available from the corresponding author upon a reasonable request.

## Supplementary Materials

The following supporting information can be downloaded at: https://www.mdpi.com/article/10.3390/medicina61040735/s1, Table S1: List of all regulated proteins identified through LC-MS/MS analysis, with label-free quantification, showing a two-fold regulation and unique peptides ≥ 2, comparing BLCA tumor tissues to control. Figure S1: Western blot validation of LC-MS/MS analysis. The Western blot results demonstrated a significant decrease in 14-3-3 beta/alpha expression in BLCA compared to the control, consistent with the findings from LC-MS/MS analysis. An asterisk (*) denotes a statistically significant difference (*p*  <  0.05).

## Abbreviations

The following abbreviations are used in this manuscript:

BLCA	Bladder Cancer
LC-MS	Liquid Chromatography/Mass Spectrometry
BCG	Bacille Calmette–Guérin
IL	Interleukin
ICIs	Immune Checkpoint Inhibitors
TME	Tumor Microenvironment
TUR-P	Transurethral Resection of the Prostate
FDR	False Discovery Rate
DRPs	Differentially Regulated Proteins
STRING	Search Tool for the Retrieval of Interacting Genes/Proteins
PPI	Protein–Protein Interaction
ICPs	Immune Checkpoint Proteins
OS	Overall Survival
MCC	Maximal Clique Centrality
CF	Complement Factor
C4BPA	Complement 4 Binding Protein Alpha
DC	Dendritic Cell
MAC	Membrane Attack Complex
EMT	Epithelial–Mesenchymal Transition
sCC	Cutaneous Squamous Cell Carcinoma
MDSC	Myeloid-Derived Suppressor Cell
TH2	T-Helper-2
Tregs	Regulatory T-Cells
NET	Neutrophil Extracellular Trap
mCRPs	Membrane-Bound Complement Regulatory Proteins
LG	Low Grade
HG	High Grade
RCC	Renal Cell Carcinoma
