# Supplementary material for: Proteomic and In Silico Analyses Highlight Complement System’s Role in Bladder Cancer Immune Regulation
Source: Medicina (Kaunas). 2025 Apr 16;61(4):735. doi: 10.3390/medicina61040735 (PMC12028855; doi:10.3390/medicina61040735)
Supplement: Supplementary file 1 [file medicina-61-00735-s001.zip › Supplementary Figure S1.pdf]

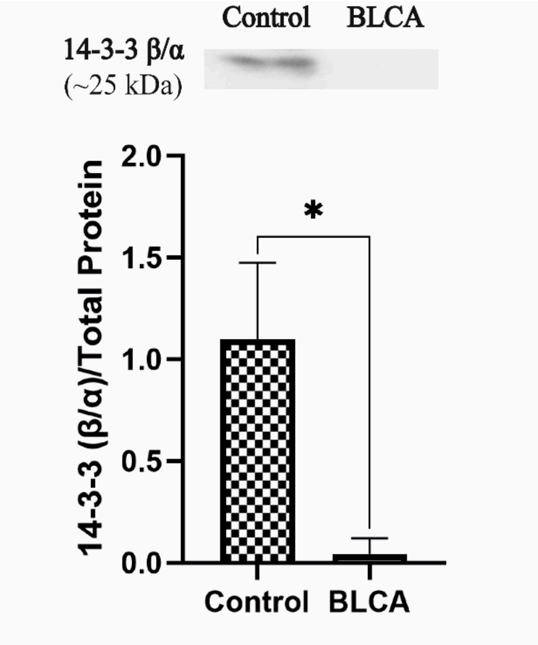

Figure S1. Western blot validation of LC-MS/MS analysis. The Western blot results demonstrated a significant decrease in 14-3-3 beta/alpha expression in BLCA compared to the control, consistent with the findings from LC–MS/MS analysis. An asterisk (*) denotes a statistically significant difference ($p < 0.05$).
